# Skin Autofluorescence, as Marker of Accumulation of Advanced Glycation Endproducts and of Cumulative Metabolic Stress, Is Not Increased in Patients with Systemic Sclerosis

**DOI:** 10.1155/2011/417813

**Published:** 2011-09-29

**Authors:** M. E. Hettema, H. Bootsma, R. Graaff, R. de Vries, C. G. M. Kallenberg, A. J. Smit

**Affiliations:** ^1^Division of Rheumatology and Clinical Immunology, Department of Internal Medicine, University Medical Center Groningen, University of Groningen, 9700 RB Groningen, The Netherlands; ^2^Department of Biomedical Engineering, University Medical Center Groningen, University of Groningen, 9700 RB Groningen, The Netherlands; ^3^Division of Endocrinology, Department of Internal Medicine, University Medical Center Groningen, University of Groningen, 9700 RB Groningen, The Netherlands; ^4^Division of Vascular Medicine, Department of Internal Medicine, University Medical Center Groningen, University of Groningen, 9700 RB Groningen, The Netherlands

## Abstract

*Objective*. To investigate whether advanced glycation endproducts (AGEs) in the skin are increased in patients with systemic sclerosis (SSc) and are related to the presence of disease-related and traditional cardiovascular risk factors. *Methods*. Skin autofluorescence, as a measure for the accumulation of AGEs, was assessed by measuring UV-A light excitation-emission matrices (AF-EEMS) in 41 SSc patients and 41 age- and sex-matched controls. Traditional cardiovascular risk factors and disease-related risk factors were recorded. *Results*. Skin AF-EEMS did not differ between SSc patients and controls (1.68 ± 0.58 a.u. versus 1.63 ± 0.41 a.u., *P* = 0.684). Skin AF-EEMS in SSc patients was associated with levels of CRP (*r* = 0.44, *P* = 0.004), Medsger's severity scale (*r* = 0.45, *P* = 0.006), and use of agents intervening in the renin-angiotensin system (*r* = 0.33, *P* = 0.027). When analysing SSc patients and controls together, in multivariate analysis, only age and use of agents intervening in the renin-angiotensin system were independently associated with AF-EEMS. *Conclusion*. These data demonstrate that skin AGEs are not increased in SSc patients.

## 1. Introduction

Vascular involvement is a key factor in major manifestations of systemic sclerosis (SSc), such as Raynaud's phenomenon (RP), myocardial dysfunction, pulmonary hypertension, and renal involvement. Microvascular involvement, in which endothelial injury is present, is the main characteristic of SSc [1, 2] Oxidative stress has been suggested as a major player in the process of endothelial dysfunction found in SSc. Endothelial damage may be induced by oxygen free radicals and reactive nitrogen species, generated locally by the inflammatory process and by periods of tissue ischemia followed by postischaemic reperfusion. This so-called ischaemic-reperfusion injury can be seen in RP [3, 4]. Increased levels of antibodies against oxidised low-density lipoproteins (LDL) [4–6] and increased serum levels of 8-isoprostane [7], being markers of oxidative stress, have, indeed, been observed in SSc.

Oxidative or carbonyl stress, leading to formation of so-called reactive carbonyl compounds, is an important source for the generation of so-called advanced glycation endproducts (AGEs) [8]. AGE generation as a result of oxidative stress has also been found in inflammatory diseases, such as rheumatoid arthritis and SLE [9–15].

Tissue autofluorescence (AF) is a marker of the accumulation of AGEs, validated in different patient groups and healthy controls [16–18]. Therefore, we assessed AGE accumulation in patients with SSc and hypothesized that AGE accumulation is increased in patients with SSc compared to healthy controls based on the presence of oxidative stress and endothelial dysfunction in SSc. We related AGE accumulation to the presence of disease-related and traditional cardiovascular risk factors.

## 2. Material and Methods

### 2.1. Patients

Forty-one patients with limited cutaneous SSc from our university medical center out-patient clinic, fulfilling the ACR criteria for SSc [19] were included. Exclusion criterium was pregnancy. Forty-one age- and sex-matched healthy subjects were recruited as controls. The local research ethics committee gave approval for the study, and informed consent was obtained from all subjects. Clinical data were obtained by chart review and questionnaires. Diabetes mellitus was defined by the criteria from the American Diabetes Association. Dyslipidemia was diagnosed if plasma cholesterol exceeded 6.21 mmol/L, LDL cholesterol exceeded 3.36 mmol/L, and triglycerides exceeded 2.26 mmol/L or when the patient used lipid-lowering drugs [20]. Hypertension could not be categorized because of frequent use in SSc patients of vasodilating agents, such as calcium channel antagonists and ketanserin for other reasons than hypertension. Creatinine clearance (CrCl) was estimated using the Cockcroft-Gault formula. Smoking status and body mass index were also recorded. The SCORE risk estimation system was used, which was originally developed to obtain an estimation of total 10-year fatal cardiovascular risk in populations, using gender, age, total cholesterol level, systolic blood pressure, and smoking status [21]. Furthermore, we assessed disease-related factors that might influence skin AF and the development of atherosclerosis. Modified Rodnan Skin Score was assessed to determine skin thickness. To assess disease activity the preliminary European Scleroderma Study Group (EScSG) activity indices (a score ranging from 0 to 10) [22, 23] and the revised preliminary SSc severity scale (Medsger's severity scale) [24] were used. Immunosuppressive therapy was recorded.

### 2.2. Assessment of Skin AF

Tissue AGEs accumulation can be assessed as skin autofluorescence (AF), following the principles of the AGE Reader, which is a validated and noninvasive technique [16, 25]. Repeated measurements on one day in controls and diabetic patients showed an overall Alman error percentage of 5%. In this study, an adapted setup of the AGE Reader was used, namely, the Excitation Emission Matrix Scanner (EEMS), which is a technique to determine skin autofluorescence (AF-EEMS), which has the additional potential to discriminate between autofluorescence spectra from different fluorophores. This technique and setup has been described [26]. Measurement was performed at a skin site of approximately 4 cm^2^ without evidence of fibrosis at the ventral site of the forearm, or other skin lesions. A series of measurements was obtained for each subject, and mean skin AF-EEMS was determined as described [14]. Skin pigmentation is also known to influence autofluorescence by light absorption. Therefore skin reflection should be >10% to perform an adequate measurement.

### 2.3. Laboratory Assessments

Lipid concentrations (total, high density lipoprotein (HDL) and low-density lipoprotein (LDL) cholesterol and triglycerides), glucose, and creatinin were measured by routine techniques. CRP was measured using in-house enzyme-linked immunosorbent assays (ELISAs) as described [27]. 

### 2.4. Statistical Analysis

Values are expressed as mean ± SD when variables were normally distributed. In case of a nonnormal distribution, values are reported as median (interquartile ranges). For comparison between groups, continuous variables were analysed by Student's *t*-test or Mann-Whitney *U* tests, as appropriate. In case of categorical variables, the chi-square test was used. The univariate correlation between AF-EEMS values and other categorical variables was assessed by Pearson's correlation coefficient in case of normal distribution. Otherwise, Spearman's correlation coefficient was used. To assess the influence of tested parameters multiple regression analysis using the backward method was performed to assess the influence of demographic variables, outcome variables, and disease-related factors on skin autofluorescence (AF-EEMS). Variables which were significantly correlated in univariate analysis were used as independent variables in the multivariate analysis.

All analyses were performed using SPSS 14.0. A two-tailed *P* value < 0.05 was considered statistically significant.

## 3. Results

### 3.1. Characteristics of Patients and Controls

Characteristics of patients and controls are presented in Tables 1 and 2. Patients and controls were similar in age and gender and regarding (family) history of CVD, presence of diabetes mellitus, and renal function. Significant differences between SSc patients and controls were present in diastolic blood pressure and lipid levels, but also in medication used. Antihypertensive agents or vasodilating agents were used in 80% of patients compared to 2% of controls. Amongst these agents, 11 patients used angiotensin-converting enzyme (ACE) inhibitors and 3 patients used an angiotensin II receptor blocker. Also, statins were used more frequently in patients. BMI was slightly higher in controls, and none of the controls had a history of cardiovascular disease. Although the presence of traditional risk factors differed significantly between patients and controls, the total cardiovascular risk, SCORE, was comparable. CRP levels were significantly increased in patients compared to controls (3.4 mg/L (IQR 1.7–7.7) versus 1.4 mg/L (0.6–2.5), *P* < 0.001). Immunosuppressive agents, such as prednisolone, methotrexate, azathioprine, and cyclophosphamide were used in 15 (37%) SSc patients.

### 3.2. Skin AF-EEMS in Patients and Controls

**Figure 1:**
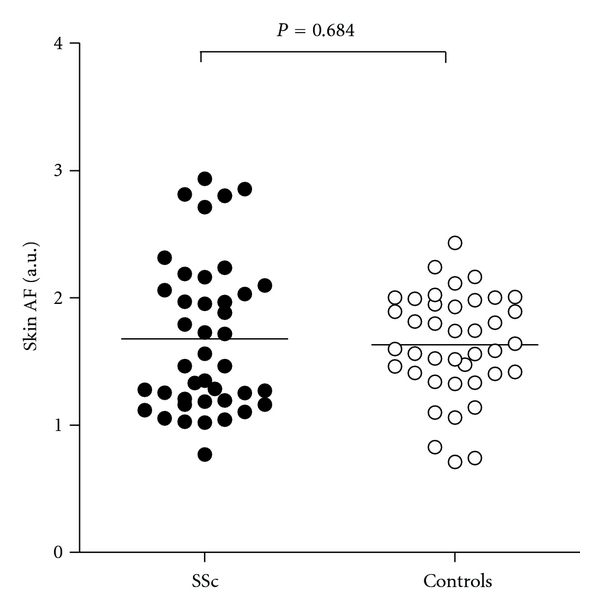
Skin autofluorescence (AF-EEMS) in patients (closed circles) and controls (open circles). The horizontal line represents mean skin AF-EEMS values.

No difference was found in AF-EEMS between patients and healthy controls (1.68 ± 0.58 a.u. versus 1.63 ± 0.41 a.u., *P* = 0.684, Figure 1). AF-EEMS was significantly higher in those with a history of CVD (*n* = 4) compared to those without a history of manifest CVD (*n* = 77) (2.22 ± 0.81 a.u. versus 1.62 ± 0.47 a.u., *P* = 0.020). In subjects using ACE inhibitors or ATII receptor blockers for hypertension or other reasons, AF-EEMS was significantly higher than in subjects not using these agents (2.01 ± 0.62 a.u. versus 1.57 ± 0.45 a.u., *P* = 0.002).

Univariate analysis between skin AF-EEMS and traditional risk factors and disease-related factors for CVD in SSc patients resulted in a positive correlation between skin AF-EEMS and CRP (*r* = 0.44, *P* = 0.004), as well as MSS (*r* = 0.45, *P* = 0.006) and use of ACE inhibitors or ATII receptor blockers (*r* = 0.33, *P* = 0.027). Univariate analysis of all subjects, patients and controls together, resulted in an association between skin AF-EEMS and age (*r* = 0.28, *P* = 0.010), CRP (*P* = 0.25, *P* = 0.026), and use of ACE inhibitors or ATII receptor blockers (*r* = 0.275, *P* = 0.013). All other clinical and biochemical variables did not show significant correlations with skin AF-EEMS.

Multivariate analysis revealed that age and use of ACE inhibitors or ATII receptor blockers were independently associated with skin AF-EEMS. Otherwise, no independent associations with skin AF-EEMS were present (Table 3).

## 4. Discussion

In this study, we demonstrated that skin AF as a marker of tissue AGE accumulation is not increased in SSc patients, while expected relations with age, prevalence of CVD, and CRP were found.

To our knowledge, only one study has been performed on the relation between AGEs and SSc. Kaloudi et al. [28] compared circulating levels of N*ε*-(carboxymethyl)lysine (CML), one of the AGEs which can be detected in vivo, in SSc patients and healthy controls by means of ELISA. They found increased CML levels in SSc patients regardless of subset, with highest the levels found in SSc patients with an “early” disease pattern in nailfold videocapillaroscopy, suggesting that AGEs are involved in SSc microangiopathy. This is in agreement with the observation that the highest values of markers of oxidative stress are seen in early stages of the disease [4]. 

Several factors may be responsible for this discrepancy. We used noninvasive skin autofluorescence measurements for the assessment of accumulation of AGEs. This technique is simple, rapid, and noninvasive. Results from this technique were found to correlate strongly with levels of AGEs measured from skin biopsies [16, 29], although this validation was not extended to patients with SSc. Also, AGE detection in tissue with long-lived (years) proteins like the skin may better reflect the chronic accumulation of AGEs than measuring AGEs from serum or plasma with a relatively short (weeks) half-life of most proteins [25]. Since disease activity in SSc patients will wax and wane, we considered noninvasive skin autofluorescence measurements more useful in these patients. Our choice for the skin autofluorescence was also supported by the increased levels found in other autoimmune diseases with intermittent disease activity like rheumatoid arthitis and SLE in which skin AF was related to integrated disease duration and damage [14, 15]. Another consideration might be that skin AF was affected in the same emission range by other fluorophores (like NADH or tryptophan) to a different extent than in other conditions. Although we cannot exclude this, the expected relations with factors like age, CRP, and history of CVD still support skin AF as a marker of AGEs.

We expected to find more AGE accumulation in SSc patients with signs of inflammation and disease severity. Compared to controls, we found higher CRP levels in SSc patients, which might suggest a more active disease although absolute levels were not increased and EscSG activity index did not reflect active disease. We found a positive correlation between skin AF-EEMs levels and MSS, a measure of activity, severity, and damage in SSc patients, suggesting a relation between AGE accumulation and disease severity. Recently, doubts have been raised by Valentini and Cerinic on the weighting of the contribution of the different organ systems in the MSS [30]. Perhaps such a disbalance in the MSS explains why MSS, and also CRP, were not found to remain as predictors of skin AF-EEMS in multivariate analysis. 

We found higher skin AF-EEMS levels in a substantial number of subjects using ACE inhibitors or ATII antagonists compared to subjects not using these agents, and their use was independently associated with skin AF-EEMS in multivariate analysis. This seems surprising because these agents have been found to reduce AGE accumulation in animal studies and in vitro studies [31–35]. In our SSc patients, these agents were used for a long period for several reasons, such as hypertension and Raynaud's phenomenon. Therefore, this treatment would have been expected to diminish AGE accumulation. We found higher skin AF-EEMS levels in subjects using these agents, but it cannot be ruled out that AGE accumulation was even more pronounced before agents intervening in the renin-angiotensin system were used.

Skin fibrosis in SSc could be a possible explanation of the lack of increase in AF-EEMS values that we had expected to occur, although we only included patients with limited cutaneous SSc, who had no significant fibrosis of the forearm. AGE accumulation and skin autofluorescence are strongly related to collagen-linked fluorescence and, thereby, to the usually very long half-time (15–20 years) of collagen. Previously reported increases in both collagen synthesis but especially increased degradation of skin collagen in SSc suggest that our results in SSc patients may be explained by accelerated skin collagen turnover [36–40]. In that case, the accelerated degradation of skin collagen could have prevented skin AGE accumulation and masked the effects of oxidative stress on AGE formation. Although assessment of skin AF-EEMS was performed at visible and palpable nonlesional skin to prevent influences by the presence of skin fibrosis, this influence cannot be ruled out completely. Also, increased collagen turnover may not be limited to affected skin only. 

 In conclusion, although oxidative stress seems present in SSc and is an acknowledged important factor in the generation of AGEs, increased levels of AGEs, as determined by skin autofluorescence, were not found. We cannot rule out that accumulation of AGEs in SSc patients was prevented by the use of ACE inhibitors or ATII receptor blockers, and by accelerated skin collagen turnover in clinically unaffected areas.

##  Conflict of Interests

R. Graaff and A. J. Smit are both founders of DiagnOptics BV, the Netherlands, manufacturing autofluorescence readers (http://www.diagnoptics.com/). The other authors have nothing to declare.

## Figures and Tables

**Table 1:** Clinical characteristics.

	Patients (*n* = 41)	Controls (*n* = 41)	*P* values
Age (years)	55.9 ± 11.0	55.4 ± 9.0	NS
Female, *n* (%)	33 (80%)	33 (80%)	NS
History of CVD, *n* (%)	4 (9%)	0	NS
Family history of CVD, *n* (%)	9 (22%)	13 (32%)	NS
Diabetes mellitus, *n* (%)	2 (5%)	0	NS
Glucose (mmol/L)	4.9 (4.3–5.4)	5.6 (5.3–6.2)	<0.001
BMI (kg/m^2^)	24.1 (21.1–25.3)	25.0 (22.2–27.1)	0.019
Creatinine (*μ*mol/L)	80 (67–93)	77 (59-84)	NS
Creatinine clearance (mL/min/1.73 m^2^)	78 ± 23	87 ± 14	NS
Current smoking	3 (7%)	0	NS
Blood pressure			
Systolic (mm Hg)	120 (11–138)	128 (118–140)	NS
Diastolic (mm Hg)	75 (70–80)	78 (72–87)	0.023
Antihypertensive or vasodilating agents	33 (80%)	1 (2%)	<0.001
ACE inhibitors or ATII receptor blockers	14 (34)	1 (2%)	<0.001
Lipid levels			
Cholesterol (mmol/L)	5.0 ± 0.9	5.8 ± 0.9	<0.001
HDL cholesterol (mmol/L)	1.3 (1.2–2.2)	1.7 (1.4–2.0)	0.025
LDL cholesterol (mmol/L)	2.9 ± 0.8	3.5 ± 0.8	0.001
Triglycerides (mmol/L)	1.4 (1.2–2.2)	1.2 (0.9–1.7)	0.035
Statin use	7 (17%)	0	0.012
Dyslipidemia, *n* (%)	18 (44)	19 (46)	NS
Aspirin use	12 (29%)	0	<0.001
SCORE, %	1.0 (0.0–2.0)	1.0 (0.0–2.0)	NS
CRP (mg/L)	3.4 (1.6–7.7)	1.5 (0.6–2.5)	<0.001

Unless stated otherwise, data are expressed as mean ± SD when normally distributed and as median (25–75%) when nonnormally distributed.

CVD: cardiovascular disease; BMI: body mass index; ACE: angiotensin-converting enzyme; ATII: angiotensin II; HDL: high-density lipoprotein; LDL: low-density lipoprotein; SCORE: systematic coronary risk evaluation; CRP: C-reactive protein.

**Table 2:** Disease characteristics and disease-related factors in SSc patients.

Characteristic	Patients, *n* (%)
Duration SSc, yr	5 (3–11)
Duration RP, yr	11 (5–23)
EScSG activity index	0.5 (0.5–2.0)
MSS	6 (5–7)
mRSS	7 (5–14)

Data are expressed as median (interquartile ranges).

SSc: systemic sclerosis; RP: Raynaud's phenomenon; EscSG: European Scleroderma Study Group; MSS: Medsger's severity scale; mRSS: modified Rodnan Skin Score.

**Table 3:** Multiple linear regression analysis with AF-EEMS as dependent variable in patients with systemic sclerosis and healthy controls (*n* = 82).

	*B*	*β*	*P* values
Constant	0.284		
Age	0.022	.400	0.006
Use of ACE inhibitors or ATII receptor blockers	0.613	.494	0.001

AF-EEMS: autofluorescence obtained by the Excitation-Emission Matrix Scanner; ACE: angiotensin-converting enzyme; ATII: angiotensin II.
